# Visceral Abdominal and Subfascial Femoral Adipose Tissue Have Opposite Associations with Liver Fat in Overweight and Obese Premenopausal Caucasian Women

**DOI:** 10.1155/2011/154672

**Published:** 2011-09-22

**Authors:** Paulo M. Rocha, José T. Barata, Cláudia S. Minderico, Analiza M. Silva, Pedro J. Teixeira, Luís B. Sardinha

**Affiliations:** Exercise and Health Laboratory, Faculty of Human Movement, Technical University of Lisbon, Lisbon, Estrada da Costa, 1495-688 Cruz Quebrada, Portugal

## Abstract

Abdominal obesity has been associated with liver fat storage. However, the relationships between other body composition depots and metabolic syndrome features with hepatic fat are still unclear. We examined abdominal and thigh adipose tissue (AT) compartments associations with liver fat in 140 overweight and obese premenopausal Caucasian women. Blood lipids and, proinflammatory and atherothrombotic markers associations with hepatic fat were also analyzed. A larger visceral AT (VAT) was related with liver fat (*P* < 0.05). Contrarily, thigh subfascial AT was inversely related to liver fat (*P* < 0.05). Increased fasting insulin, triglycerides, PAI-1 concentrations, and a higher total-cholesterol/HDL-cholesterol ratio were also associated with hepatic fat, even after adjustment for VAT (*P* < 0.05). Thigh subfascial adiposity was inversely associated with liver fat, suggesting a potential preventive role against ectopic fat storage in overweight and obese women. These results reinforce the contribution of an abdominal obesity phenotype associated with a diabetogenic and atherothrombotic profile to liver lipotoxicity.

## 1. Introduction

Obesity-related comorbidities seem to be more closely related to body fat distribution (e.g., upper versus lower, visceral versus subcutaneous, and truncal versus peripheral) rather than the total amount *per se *[1]. Abdominal obesity is a relevant predictor of a higher metabolic risk, assuming that insulin resistance (IR) is the common link between visceral adiposity and dyslipidemia [2–4], type 2 diabetes mellitus (DM) [5, 6], liver fat storage [7], hypertension [8], and other cardiovascular diseases (CVD) [8–10]. Two major pathophysiological hypotheses have been proposed to explain the dysmetabolic milieu observed in abdominal obese individuals. It has been proposed that neuroendocrine perturbations, mediated by hypothalamic-pituitary-adrenal (HPA) axis stimulation, are responsible for IR and abdominal obesity [11, 12]. Alterations in cortisol secretion, inhibition of steroid and growth hormones production, and stimulation of sympathetic nervous centers are some of the dysfunctions which may precipitate metabolic disturbances [12]. Conversely, according to the “portal hypothesis,” the increased lipolytic activity in visceral adipocytes leads to an augmented release of free fatty acids (FFA) into portal circulation, promoting liver fat storage that is accompanied by hepatic metabolism disturbances and IR [6, 13, 14]. 

In this context, abdominal obesity has been associated with ectopic fat storage, defined as fat accumulation outside “classical” depots such as heart, skeletal muscle, pancreas, and liver [15]. Liver fat is associated with obesity, increased concentrations of plasma FFA, as well as with the IR degree, both in obese and type 2 DM patients [14, 16, 17]. Furthermore, a lower liver-to-spleen ratio (LSR), a reliable index of liver fat [18], has been independently associated with visceral adiposity [7, 19, 20], hepatic IR [6, 17, 19, 21], dyslipidemia, and several other metabolic syndrome features [6, 7, 19, 22]. Additionally, hepatic steatosis has also been associated with IR and major CVD risk factors due to a combination of abnormalities including increased liver FFA influx and synthesis, decreased FFA oxidation, increased very low-density lipoprotein (VLDL) particles secretion, and a low-grade chronic proinflammatory state [21, 23]. 

Although evidence has highlighted the independent contributions of both visceral adiposity and liver fat to an increased metabolic risk in obese and type 2 DM patients, it is not totally clear if liver fat is additionally associated with other specific inflammatory and atherothrombotic markers [6, 7, 19, 22]. On the other hand, despite the recognized abdominal obesity relevance to ectopic fat storage, little is known about the relationships of other fat compartments, such as thigh AT, with liver fat, and consequently with hepatic steatosis. In fact, only one study developed in type 2 DM patients has reported that thigh subfascial AT was associated with both liver fat and IR [6] features. Therefore, based on previously defined criteria [18], the current study examined the independent associations of abdominal and thigh AT compartments with hepatic fat in overweight and obese premenopausal Caucasian women. Additionally, this study investigated the associations of liver fat with metabolic, proinflammatory, and atherothrombotic risk factors.

## 2. Materials and Methods

### 2.1. Subjects

Participants in this investigation were 140 premenopausal overweight and obese Caucasian women, recruited from the Lisbon (Portugal) area by public advertisements. Participants were part of a 2-year weight management program, as described elsewhere [24]. Study inclusion criteria were the following: subjects could not be pregnant or trying to become pregnant, >24 years, >24.9 kg/m^2^  body mass index (BMI), were not under any medication that could affect weight, body composition or liver metabolism, had no clinical or laboratory evidence of liver or spleen disease, and had no history of cancer in the last five years. Exclusion criteria were ongoing hormonal medication, history of CVD, stroke, hypertension, type 2 DM, Cushing syndrome, hormonal dysfunction, resting and exercise abnormal electrocardiograms, as well as the presence of drinking habits. Subjects that were undertaking oral medication to treat hypertriglyceridemia, hyperglycemia, or hypercholesterolemia were also excluded. All subjects were informed about the purpose, nature and study design before giving their full written consent to participate. The study protocol was performed according to the principles of the Helsinki Declaration and was approved by The Human Subjects Institutional Review Board of the Faculty of Human Movement, Technical University of Lisbon.

### 2.2. Body Composition Assessments

#### 2.2.1. Anthropometric Variables

Height was measured to the nearest 0.1 cm with a stadiometer (Seca, Hamburg, Germany). Body weight was measured to the nearest 0.01 kg on a previous calibrated scale after removing shoes and heavy clothing. Abdominal sagittal diameter (SD), waist circumference (WC), and hip circumference (HC) measurements were performed according to Lohman et al. [25] procedures. Both WC and HC measurements were taken with a stiff fibreglass tape to the closest 0.1 cm. All anthropometric variables were measured by trained technicians and repeated 3 times, with the mean value being used. BMI was calculated as weight divided by height squared (kilograms per square meter), and waist-to-hip ratio (WHR) was defined as the WC divided by HC.

#### 2.2.2. Dual Energy X-ray Absorptiometry (DXA)

Trunk fat mass (TFM), total body fat mass (TBFM), and total body lean mass (TBLM) were measured by a pencil beam mode DXA (QDR-1500 Hologic, Waltham, Mass, USA). All measurements were made with volunteers in the supine position with their arms separated from their trunk. The same technician performed the all the scans and completed the analysis according to the operator's manual. The intraobserver coefficient of variation (CV) for TBFM and TBLM was 2.0% and 1.7%, respectively. A 0.5% technical error for %TBFM was obtained as estimated in 2 repeated measures performed on 10 subjects.

#### 2.2.3. Measurement of Abdominal Adipose Tissue Distribution

With the subjects supine and arms extended above their head, a single cross-sectional CT (Somaton Plus; Siemens, Sorheim, Germany) L4-L5 intervertebral space image was acquired to measure abdominal AT compartments, as described elsewhere [1]. All images were obtained using 120 kVp, 480 mA, and 512 × 512 matrix with a 48-cm field of view. Total abdominal adipose tissue (TAAT), abdominal subcutaneous adipose tissue (Ab SAT), superficial and deep Ab SAT, and visceral AT (VAT) areas were measured. The boundary between VAT and Ab SAT was defined using the abdominal and oblique muscles in continuity with the deep fascia of the paraspinal muscles and the anterior aspect of the vertebral body [26]. The subcutaneous fascia was used to differentiate Ab SAT into its superficial and deep compartment [1].

#### 2.2.4. Measurement of Thigh Adipose Tissue and Muscle Distribution

Using the same scan parameters, contiguous 7-mm-thick cross-sectional images of both legs were obtained between the inferior ischial tuberosity and the superior border of the patella. Total thigh adipose tissue (TTAT), total thigh subcutaneous AT (TTSAT), thigh subfascial AT (TTSFAT), and muscle tissue areas and attenuations were measured. The tissues volumes (cubic centimetres) identified in each image were calculated by multiplying the image thickness (7 mm) by the tissue area (square centimetres). Thigh AT volume (litters) was then converted to mass units (kilograms) multiplying the volume by the assumed constant of fat density (0.92 kg/L) [27]. Total thigh muscle mass was also calculated multiplying its volume by the constant density assumed for AT-free skeletal muscle (1.04 kg/L) [27]. From the thigh scans performed, it was selected a single slice located at the mid-point distance between both anthropometric markers previously described to image mid-thigh AT and muscle tissue distribution.

#### 2.2.5. Measurement of Liver Fat

A 7-mm-thick cross-sectional image at T11-T12 intervertebral space was acquired to measure both liver and spleen CT attenuations, which were determined by calculating the mean Hounsfield units (HUs) of three regions of interest (ROI) (liver ROI had *∼*120 mm^2^, located 2 in right lobe and 1 in left lobe; spleen ROI had *∼*75 mm^2^). As previously described [6], all ROI were consistently selected in peripheral parenchyma areas, away from artefacts, major blood vessels and other areas of inhomogeneity. The ratio of mean liver to spleen attenuation values, defined as liver-to-spleen ratio (LSR), has been defined as reliable index of liver fat [18]. Fatty liver was present when LSR < 1 [6].

#### 2.2.6. Measurements Repeatability

The repeatability for abdominal and thigh adipose and muscle tissue compartments was calculated in 30 women chosen randomly from all subjects. One technician performed the repeated analyses on the same images, separated by 3 months. The intraobserver coefficient of variation (CV) for VAT and TAAT was, respectively, 0.9% and 0.7%. For Ab SAT, deep and superficial Ab SAT, the intra-observer CV was, respectively, 0.8%, 2.8%, and 3.1%. For mid-thigh muscle tissue, total mid-thigh AT and subfascial AT (SFAT), the intra-observer CV was 0.1%, 0.4%, and 2.5%, respectively. Repeatability for liver and spleen measurements was also examined; the CV found for LSR was 2.2%.

#### 2.2.7. Image Analysis

Based on image morphology, CT data were analysed by specific software (Slice-O-matic, Version 4.2, Tomovision, Montreal, Canada). A combination of watershed techniques and edge detection filters was employed. Standard HU ranges for adipose tissue (−190 to −30 HU) and skeletal muscle (0 to +100 HU) were used to compute the tissue segmentation [28]. It was also measured, in both legs, the high-density (30 to 100 HU) and low-density muscle areas (0 to 30 HU) [28]. Thigh fascia was used as boundary to demarcate the subcutaneous from subfascial AT [28].

### 2.3. Blood Analysis

After a 12-hour overnight fast, venous blood samples were collected at antecubital vein. Samples were centrifuged at 4°C, and plasma was stored at −70°C until analysis. Measurement of triglycerides (TG), uric acid, total cholesterol (TC), low-density lipoprotein cholesterol (LDL-C), and high-density lipoprotein cholesterol (HDL-C) was made by enzymatic colorimetric methods. Fasting insulin was determined by electrochemiluminescence immunoassay (ECLIA), glycemia was assessed by hexokinase method and interleukin-6 (IL-6) was measured by chemiluminescence immunoassay. Tumor necrosis factor-alpha (TNF-*α*) was measured using a high-sensitivity enzyme-linked immunosorbent assay (ELISA) principle. Plasminogen activator inhibitor-1 (PAI-1) was measured in iced citrated plasma using the Coatest PAI method (enzyme immunoassay—EIA), and fibrinogen concentrations were measured by clotting time. Hemoglobin A1c (Hb A1c) was determined by high-pressure liquid chromatography (HPLC). Serum adiponectin and leptin concentrations as well as urine cortisol were measured by radioimmunoassay (RIA).

Microalbuminuria, C-reactive protein (CRP), apolipoprotein A1 (apo A1), and apolipoprotein B100 (apo B100) plasma concentrations were measured by high-sensitivity particle-enhanced turbidimetric assay. Alanine aminotransferase (ALT) and aspartate aminotransferase (AST) were determined by a kinetic method. Samples were measured in duplicate, and the average of the two values was used in the statistical analyses. All blood samples were taken during the follicular stage of menses.

### 2.4. Blood Pressure

At the same day of body composition measurements, systolic and diastolic blood pressures (BP) were measured in seated position with a semiautomatic oscillometric recorder (Dinamap, Critikon, Tampa, FL) after a 5-minute rest period. A suitable cuff size was applied to participant's upper arm at the heart level, and the mean of three measurements in each arm, separated by 1-minute time lapse, was calculated. Previously to the baseline measurements, all subjects were clinically evaluated by the program physician who was responsible for the final (clinical) decision related with the inclusion or exclusion of each subject in the study sample. Regarding hypertension criteria, all subjects included in our study were not hypertensive. However, at the same day of body composition measurements, one subject revealed a mean systolic blood pressure of 175 mmHg (DBP: 93 mmHg) while in another subject was measured a mean diastolic blood pressure of 105 mmHg (SBP: 150 mmHg). In order to confirm the nonhypertensive status previously assigned, both subjects repeated the assessments in another day of the same week, and the non-hypertensive status was confirmed. For the study purposes, since all laboratory measurements were specifically made at the same day, we included both subjects in the study and in data analysis.

### 2.5. Premenopausal and Physical Activity Status

The study physician, based on her menstrual history, determined the premenopausal status of each study volunteer; the premenopausal stage was considered if women reported regular menstrual cycles. Daily physical activity status was assessed by proportional actigraphy which measure the time spent in different daily activities based on the number of counts per minute (Computer Science and Applications, Model AM7164, FL, USA).

### 2.6. Drinking Habits

The amount of alcohol intake was determined using a questionnaire containing information on the daily alcohol intake of various alcoholic beverages. One alcoholic unit corresponded to a glass of wine, a can of beer, or a measure of spirits, and contains 12-13 g of ethanol.

### 2.7. Statistical Analyses

Unless otherwise is indicated, data are presented as means ± SD. Control for normality and homoscedasticity of study variables was conducted. When necessary, log transformations were used to normalize distributions. Multiple linear regressions, adjusted for age and BMI, were performed to study the independent associations of liver-to-spleen ratio with major metabolic syndrome features, and inflammatory and atherothrombotic disturbances. Adjusting for the same variables, independent relations of LSR with anthropometric markers, abdominal and thigh adipose and muscle tissue compartments were also studied. Additional multiple linear regressions, adjusted for age, fat mass, and VAT, were performed to study the associations between LSR and all the metabolic syndrome profile markers.

In order to facilitate the comparisons of the results obtained in the multiple linear regression models, standardized beta coefficients were presented. To determine how much the independent variables were linearly related to one another, the multicolinearity was studied by statistic tolerance (1-R^2^), being the stability of the regression model disturbed by multicolinearity if tolerance was inferior to 0.1. Statistical significance was set at *P* < 0.05. Statistical analyses were performed using the SPSS version 13.0 for Windows (SPSS, Chicago, Ill, USA).

## 3. Results

Most subjects were obese. Both body weight and BMI revealed a wide range of variation (mean ± sd: 78.1 ± 1.0 kg, range: 59.1–107.8 kg; 30.4 ± 0.3 kg/m^2^, 25.1–45.2 kg/m^2^, resp.). The sample age varied between 25 and 49 years (38.3 ± 0.5 yrs). At baseline, very few subjects included small amounts of physical activity in their daily routine. In fact, the majority of the study subjects included were sedentary, since 94.4% of the daily time was spent in activities with intensities under 100 counts per minute (data not shown). Subject's anthropometric and body composition characteristics are presented in Table 1. Similarly to BMI, both abdominal and thigh AT compartments revealed a wide variation. An increased WC was observed in 43.9% of the subjects [29]. VAT was the minor constituent of abdominal AT area (23.6%). Superficial and deep Ab SAT areas were similar, comprising each one, approximately, half of total Ab SAT depot. While TTAT mass represented 57.9% of total thigh mass, LDM was the smallest compartment (19.8%) of mid-thigh muscle area. The fatty liver prevalence observed in our sample of overweight and obese premenopausal women was 2.9%. 

Metabolic syndrome features are presented in Table 2. According to the Adult Treatment Panel (ATP) III criteria [29], hypertriglyceridemia was found in 22.3% of the subjects, and hypertension assumed a 21.4% prevalence rate. In our sample, lower HDL-C concentrations were found in 44.5% of the subjects, and 9.3% of the women met the ATP III criteria for metabolic syndrome. However, only 0.7% of the study subjects revealed hyperglycemia.

Age was not associated with LSR (*β*  = 0.118, *P* > 0.05). On the contrary, both weight and BMI were inversely associated with LSR, even when adjusting for age (*β* = −0.235, *P* < 0.01; *β* = −0.225, *P *< 0.01, resp.). The results of simultaneously entering each anthropometric and body composition marker to predict LSR, adjusting for age and BMI, are shown in Table 3. Higher SD values were independently associated with a lower LSR representing an increased liver fat storage. Similar associations were observed when using liver attenuation as the dependent variable. Despite not significant, WHR, as well as total body and trunk fat mass revealed an inverse association trend with liver fat. In further analysis, after adjusting for HC, a larger WC was related with a lower LSR (*β* = −0.203, *P *< 0.05).

In Table 4 are presented the independent associations of abdominal AT depots and thigh body composition compartments with LSR, after adjustment for age and BMI. Higher VAT areas were associated with a lower LSR. On the contrary, a higher thigh SFAT area was related with a higher LSR. These associations remained significant when using liver attenuation as a dependent variable and adjusting for the same confounders. Furthermore, thigh SFAT remained positively associated with LSR, independently of WC (*β* = 0.166, *P *< 0.05).

Independent associations of metabolic syndrome features, inflammatory and atherothrombotic risk factors with liver fat, adjusting for age and BMI, are presented in Table 5. Higher fasting insulin, TG, liver transaminases, PAI-1, and uric acid concentrations, as well as higher TC/HDL-C and LDL-C/HDL-C ratios were associated with a lower LSR. When adjusted for VAT, these health risk factors remained significantly related with LSR (*P *< 0.05). The explained variance for each metabolic risk factor studied to LSR varied between 3.6% and 24.8%, showing higher values for liver transaminases, TC/HDL-C ratio, and fasting insulin. When adjusting for age and BMI and using liver attenuation as dependent variable, similar associations to those described earlier were found, except for fasting insulin and uric acid (*P *> 0.05). 

## 4. Discussion

Our primary findings were that, in a sample of overweight and obese premenopausal women, a higher thigh SFAT area was associated with a higher LSR, representing a lower liver fat storage, independently of age and BMI. Additionally, we found that for a given WC, increased thigh SFAT areas were also significantly related with higher LSR values. To our knowledge, these associations between thigh SFAT and both LSR and liver attenuation are novel observations that may suggest an indirect preventive role of this thigh AT depot against ectopic liver fat storage, and therefore, against hepatic steatosis, in overweight or obese premenopausal women without type 2 DM. It has been suggested that femoral-gluteal AT compartments may function as a “sink” for circulating FFA [30]. When compared with visceral adipocytes, these thigh adipocytes are less sensitive to stimulated lipolysis and reveal a relatively higher lipoprotein lipase activity, important in FFA uptake from the circulation [31]. These metabolic characteristics may prevent liver lipotoxicity and counteract the inevitable physiologic cascade observed in abdominal obese subjects, responsible for IR and other secondary metabolic disturbances, such as a multiple proinflammatory cytokine response. In this context, several studies have been reporting that peripheral fat mass (PFM), measured by DXA (unable to differentiate the different thigh adipose tissue compartments), is an independent predictor of a lower cardiovascular risk [32, 33]. This potential protective role of PFM in metabolic disturbances and atherogenicity may be, in part, explained by adiponectin insulin sensitizing effects [34]. In fact, it has been suggested that specifically the thigh subcutaneous AT, a major contributor for the circulating adiponectin, could mediate these counteracting effects [35]. Although little is known regarding the necessity of making a clear distinction between the metabolic activity and role of the femoral subcutaneous and subfascial fat depots, it was already assumed that subfascial thigh fat, representing the intermuscular (within muscle fibbers) fat deposition [6], is characterized by a different lipolysis rate and cytokine secretory profile, compared to subcutaneous femoral fat [1, 35]. 

In our study developed in overweight and obese premenopausal women, beside the associations found suggesting that thigh subfascial AT may confer a metabolic protection against detrimental ectopic fat storage in the liver, although not significant, a similar trend was observed between both total thigh AT mass and subcutaneous AT and LSR. In this context, the metabolic differences previously described between both thigh adipose tissue compartments might underlie different mechanisms that could interfere with the relations found in this study.

Other studies conducted with type 2 DM patients have also reported that thigh SFAT is associated with hepatic fat [6] and IR [6, 28]. Indeed, in a recent study developed with 83 type 2 DM patients, it was observed that fatty liver was inversely related with femoral subfascial AT and with visceral adiposity [6], independently of the effects of VAT and BMI. More than interpreting these results as an evidence suggesting a causative role of thigh SFAT to fatty liver pathogenesis, the authors have proposed that SFAT together with fatty liver are special adiposity depots related with IR pathogenicity in type 2 DM. These results obtained in type 2 DM patients contrast with our observations in overweight and obese premenopausal Caucasian women, suggesting that this body composition area warrants more research in order to clarify the possible underlying mechanisms. However, one possible explanation for the differences found between both studies might be related with the fact that the unfavourable metabolic profile normally present in type 2 DM patients contrasts with a relative healthy metabolic pattern found in our sample of overweight and obese premenopausal women (only 9.3% of the women met the ATP III criteria for metabolic syndrome).

The role of abdominal obesity on ectopic liver fat storage and the concomitant metabolic abnormalities was already addressed [6, 7, 36, 37]. In a study with 144 patients with hepatic steatosis, clinically characterized by hepatocyte fat infiltration and often described as fatty liver, BMI was the unique independent predictor of the steatosis degree [38]. Another two studies have also reported that, both in obese patients [39] and in living liver donors [40], BMI was associated with the steatosis severity. In our study with overweight and obese premenopausal Caucasian women, we found that, independently of age, a higher weight, BMI and sagittal diameter (but not WC) were associated with a lower LSR. Despite the nonsignificant association verified between LSR and WC when adjusting for age and BMI, in fact, WC has been described as a better predictor of VAT rather than SD. One explanation for this observation may be related with the measurement procedures, which may vary according to the method used; when WC is measured by Lohman et al. [25] procedures, it is selected the narrowest circumference in abdominal area—procedure adopted in out study. Contrarily, according to the National Health and Nutrition Examination Survey-NHANES III guidelines, WC is measured immediately above the iliac crests increasing the absolute mean values registered. In our study, despite the nonassociation verified between LSR and WC when adjusting for age and BMI, there is a trend that could be significant if adopted another measurement procedure. The observation made in our study that, for a given WC, increased thigh SFAT areas were also significantly related with a higher LSR seems to reinforce the importance of taking into account the WC in this analysis. In addition, another reason to justify the inclusion of WC in these statistical analysis procedures is related with the fact that WC seems to be clinically easer to measure rather than SD and VAT (usually implying specific equipment and more complex procedures).

On the other hand, in a study with 221 chronic hepatitis C patients [41], VAT, rather than BMI, was a significant predictor of hepatic steatosis. In fact, abdominal obesity markers, such as WC [17, 20], WHR [20, 42], VAT [4, 7], VAT/TAAT ratio [4, 7], and Ab SAT [20] have been markedly associated with liver fat. In our study, after adjustment for HC, a larger WC was related with liver fat storage. Additional adjustments for age, BMI, and thigh SFAT revealed that a higher VAT area was independently associated with a lower LSR (*β*  = −0.250, *P *< 0.05), emphasizing the abdominal obesity phenotype relevance to liver lipotoxicity [15] even in a sample of overweight and obese premenopausal women. This obesity phenotype relevance, additionally corroborated in our sample by the fact that when adjusting for age, BMI, and VAT the thigh SFAT did not remain significantly related with LSR (*β*  = 0.053, *P* > 0.05), was, in fact, already suggested in a previous study with type 2 DM patients [6] and in other recent study reporting that surgical VAT removal could reverse hepatic IR [43]. The link between abdominal adiposity and liver fat storage may be explained by the fact that FFA are more easily mobilized from visceral AT rather than Ab SAT depots, draining directly into the liver via portal circulation [44]. The increased FFA liver influx may induce hepatic steatosis that might be responsible for other metabolic disturbances, such as increased liver FFA and TG-rich lipoproteins synthesis, adipocyte proliferation failure, and insufficient hepatocyte FFA oxidation [15, 19, 45]. In addition, liver lipotoxicity may be accompanied by a low chronic inflammatory state, which can promote a future progression to nonalcoholic steatohepatitis (NASH) [45]. Despite evidence has been demonstrating the VAT-derived FFA contribution to these pathophysiologic cascade, a recent overview have also highlighted the role of FFA released from abdominal subcutaneous adipocytes into systemic circulation to these hepatic disturbances [46]. In this context, the results of our study with premenopausal overweight and obese women are consistent with some emerging observations [7], suggesting that liver fat is strongly associated with abdominal obesity and can also independently reflect an unfavourable metabolic syndrome profile. 

Indeed, we observed that higher insulin, TG, liver transaminases, PAI-1, and uric acid concentrations were independently associated with a lower LSR. Furthermore, higher TC/HDL-C and LDL-C/HDL-C ratios were also related with lower LSR values. These metabolic markers remained significantly associated with liver fat, independently of VAT (data not shown). Despite some evidence have been proposing that hepatic fat storage is normally preceded by VAT accumulation, our results are consistent with other observations reporting that liver fat remains associated with metabolic syndrome features independently of total and visceral adiposity [22, 47]. In this sense, these results suggest that hyperinsulinemia, hypertriglyceridemia and hypercholesterolemia are relevant to the metabolic cascade that mediates liver disturbances in overweight and obese premenopausal women. Other studies developed with both insulin-sensitive and insulin-resistant subjects have also reported that liver fat was associated with IR markers [7] and TG concentrations [5, 7]. Another study developed with type 2 DM patients reported that the presence of fatty liver was associated with a higher degree of IR and dyslipidemia [6]. Hepatic steatosis has also been associated with dyslipidemia, hyperinsulinemia, and IR not only in obese subjects but also in lean subjects without glucose intolerance [21]. Although the role of diabetes in hepatic steatosis and in its progression to NASH still remains unclear [45], the NHANES-III reported that simple IR features, such as fasting insulin, Hb A1c, and C-peptide concentrations, as well as abdominal obesity markers were independently associated with ALT concentrations, the most sensitive indicator of liver cell integrity. In fact, increased liver transaminases concentrations are associated with obesity severity and can also predict the liver injury degree [39, 48]. On the other hand, it is noteworthy that hyperinsulinemia seems to play a key role in FFA metabolism and may inhibit hepatocyte mitochondrial beta-oxidation, which can additionally contribute to liver lipotoxicity. The inverse associations of both PAI-1 and uric acid with LSR observed in our study emphasize the ectopic liver fat storage relevance to inflammatory and atherothrombotic metabolic syndrome disturbances in overweight and obese premenopausal Caucasian women. 

Despite several studies have been demonstrating that metabolic disturbances are associated with ectopic liver fat storage, including ours, Després et al. [49] have suggested that it might be the lack of or a dysfunctional subcutaneous adipose tissue that may be responsible for the increase in ectopic fat deposition in the liver, heart, skeletal muscle and pancreas which further increase the cardiovascular disease and type 2 DM risk, rather than the inverse way. In order to address this hypothesis, we investigate in our sample of overweight and obese women the independent contribution of LSR to each one of the metabolic syndrome markers studied. Similarly to results previously reported, LSR was associated with fasting insulin (*β* = −0.173, *P* < 0.001), triglycerides (*β* = −0.205, *P* < 0.05), TC/HDL-C (*β* = −0.212, *P* < 0.01), and LDL-C/HDL-C (*β* = −0.469, *P* < 0.05) ratios, and with both ALT (*β* = −0.354, *P* < 0.05) and AST concentrations after adjustment for age, total fat mass, and VAT. The explained variance of LSR to each metabolic risk factor studied varied between 15.5% and 29.2%, showing higher values for TC/HDL-C and LDL-C/HDL-C ratios, liver transaminases, and fasting insulin, respectively. Despite the independent associations verified between LSR and both PAI-1 (*β*  = −0.232, *P *< 0.01) and uric acid (*β*  = −0.200, *P* < 0.05) concentrations when controlled for age and BMI, they did not remain significant in this new treatment. The similarity of the independent associations verified between LSR, and each one of the metabolic syndrome markers studied in our sample suggests that a biological continuum may underlie the relations making hard to discriminate a cause-consequence interpretation.

The role of some adipocytokines, such as leptin and TNF-*α* in hepatic steatosis has also been increasingly studied. Recent studies have reported that leptin can mediate lean body tissues protection against lipotoxic damage [50], being also relevant in lipogenesis blocking, and in muscle insulin-sensitization and fatty acid oxidation enhancement [50]. However, hyperleptinemia, commonly present in visceral obese patients, may aggravate IR and promote liver fat storage. On the other hand, inflammatory cytokines, such as TNF-*α* and IL-6, often overexpressed in obese patients or overweight subjects with type 2 DM, have also been associated with liver fat and NASH pathogenesis [45]. Contrarily to the observed in a previous study with type 2 DM patients [6], in our study with premenopausal overweight and obese women, both LSR and liver attenuation were not associated with leptin, IL-6, TNF-*α*, and with any other specific inflammatory and thrombotic risk factors studied. Similar results were obtained when we analysed the independent contribution of LSR to both metabolic markers, after adjusted for age, total fat mass, and VAT (data not shown). These results maybe be explained by relatively “healthy” metabolic profile found in our sample when compared to the unfavourable profile usually found in type 2 DM patients.

The CT abdominal and thigh adipose and muscle tissue assessments, as well as the broad list of metabolic features measured and the considerable sample size (*n* = 140) are some of the strengths of this study. Additionally, participants were counseled to refrain from exercise at least 48 hours prior to blood sampling, avoiding metabolic acute exercise interferences. However, there are limitations in our study that warrant mention. First, it is noteworthy that liver attenuation obtained by CT cannot quantify absolute liver fat because attenuation of each voxel is a function of its lipid, lean tissue, and water composition. Therefore, variations in each one of the components may change the resultant attenuation, adding difficulties in data interpretation. Second, despite careful attention in ensuring bloods samples were taken during the follicular stage of menses, we did not control the diet prior blood sampling. Since lipid levels of liver and muscle can present slightly acute differences depending on diet, this issue may also slightly influence CT attenuations. Third, the low prevalence of women (2.9%) presenting a fatty liver found (as defined by a LSR < 1) in our study is also an important issue to consider since the observations made in our study with overweight and obese premenopausal women can not be extrapolated for obese or diabetic patients presenting a dysmetabolic milieu totally different from relatively healthy metabolic subjects. Finally, it is well established that men and women (before menopause) present different body composition patterns. Adipose tissue accumulation in overweight and obese men tends to be concentrated in the abdominal area whereas women tend to accumulate fat in gluteal-femoral area. It is also known that these different adipose tissue depots present different metabolic characteristics that might be responsible for the body composition differences observed. In this sense, in our study with overweight and obese premenopausal women, the associations observed cannot be extrapolated for male subjects, especially those related with the possible preventive role of thigh SFAT to liver lipotoxicity since several metabolic characteristics present in female gluteal-femoral adipocytes seem to be reduced in male adipocytes.

In summary, contrarily to previous observations made in obese type 2 DM patients, thigh subfascial adiposity was independent and inversely associated with liver fat in overweight and obese women, suggesting that this thigh AT compartment may play a preventive role against detrimental liver ectopic fat storage. Conversely, our results emphasize the contribution of a higher BMI and visceral AT, especially if associated with hyperinsulinemia, dyslipidemia, and an inflammatory and atherothrombotic profile to the metabolic cascade that dialectically interacts with liver lipotoxicity in overweight and obese premenopausal Caucasian women.

##  Conflict of Interest

The authors declare that they have no conflict of interests to report in this research.

## Figures and Tables

**Table 1:** Subjects anthropometric data, body composition data (DXA), fat and muscle distribution data (CT), and liver and spleen variables.

	Mean ± SD	Range
Anthropometric data		
WC, cm	87.2 ± 0.8	71.1–123.4
Waist-to-hip ratio	0.78 ± 0.01	0.64–0.99
Sagittal diameter, cm	20.5 ± 0.2	16.3–31.0
Fat mass		
TFM, kg	18.0 ± 0.4	9.4–32.3
TBFM, kg	36.1 ± 0.7	23.5–60.3
TBLM, kg	41.2 ± 4.62	29.7–55.6
Abdominal adipose tissue		
TAAT, cm^2^	470.9 ± 12.1	211.9–910.8
VAT, cm^2^	111.3 ± 4.3	24.9–266.8
Ab SAT, cm^2^	353.6 ± 9.1	145.0–633.4
Superficial, cm^2^	192.2 ± 5.0	90.6–384.2
Deep, cm^2^	161.8 ± 5.4	54.7–344.9
Thigh compartments		
Thigh AT, cm^2^	270.7 ± 6.9	132.9–509.1
Thigh SAT, cm^2^	261.6 ± 6.8	129.4–501.6
Thigh SFAT, cm^2^	3.5 ± 0.2	1.0–11.9
Muscle, cm^2^	234.3 ± 2.6	176.3–324.7
Muscle Attenuation, HU	44.0 ± 0.3	33.4–51.7
HDM, cm^2^	189.3 ± 2.3	145.7–264.4
LDM, cm^2^	32.8 ± 0.9	15.7–80.0
TTAT, kg	8.4 ± 2.1	4.0–14.8
TTSAT, kg	7.9 ± 2.1	3.8–14.0
TTSFAT, kg	0.6 ± 0.2	0.3–1.5
TTMT, kg	6.1 ± 0.9	4.4–10.3
Liver and spleen variables		
Liver attenuation, HU	59.8 ± 0.8	−5.6–71.0
Spleen attenuation, HU	46.4 ± 0.4	34.0–57.5
LSR	1.30 ± 0.02	−0.11–1.82

Values are means ± SD. WC, waist circumference; TFM, trunk fat mass; TBFM, total body fat mass; TBLM, total body lean mass; TAAT, total abdominal adipose tissue; VAT, visceral adipose tissue; Ab, abdominal; SAT, subcutaneous adipose tissue; Thigh SAT, mid-thigh subcutaneous adipose tissue; SFAT, subfascial mid-thigh adipose tissue; HU, Hounsfield units; HDM, mid-thigh high-density muscle; LDM, mid-thigh low-density muscle; TTAT, total thigh adipose tissue; TTSAT, total thigh subcutaneous adipose tissue; TTSFAT, total thigh subfascial adipose tissue; TTMT, total thigh muscular tissue; HU, Hounsfield units; LSR, liver-to-spleen ratio.

**Table 2:** Metabolic syndrome characteristics of the study population (*n* = 140).

	Mean ± SD	Range
Glucose homeostasis		
Fasting insulin, *μ*IU/mL	8.2 ± 0.3	2.40–17.9
Fasting glycaemia, mg/dL	89.5 ± 0.7	73.0–113.0
Hb A1c, %	4.9 ± 0.4	4.0–7.0
Lipid profile		
TC, mg/dL	194.7 ± 3.9	101.0–307.0
HDL-C, mg/dL	54.1 ± 1.1	29.0–91.0
LDL-C, mg/dL	123.5 ± 3.6	45.0–255.0
TC/HDL-C ratio	3.7 ± 1.1	2.0–9.6
LDL-C/HDL-C ratio	2.4 ± 0.1	0.9–6.1
Apo A1/Apo B100 ratio	1.7 ± 0.1	0.8–3.3
Triglycerides, mg/dL	101.5 ± 4.9	32.0–329.0
Blood pressure		
Systolic, mm Hg	120.7 ± 1.4	90.0–175.0
Diastolic, mm Hg	75.8 ± 0.9	50.0–101.0
Liver enzymes		
ALT, IU/L	18.2 ± 0.5	9.0–43.0
AST, IU/L	16.2 ± 0.6	5.0–44.0
Inflammation		
hs-CRP, mg/dL	0.45 ± 0.03	0.03–1.14
Cytokines		
IL-6, pg/mL	10.3 ± 0.6	0.8–31.5
TNF-*α*, pg/mL	3.8 ± 0.2	0.9–14.1
Hypercoagulation		
PAI-1, ng/mL	21.2 ± 2.0	1.0–100.0
Fibrinogen, mg/dL	369.4 ± 6.5	201.0–552.0
Adipokines		
Leptin, ng/mL	32.9 ± 43.3	0.9–167.4
Adiponectin, ng/mL	9.2 ± 6.4	2.9–41.0
Urine		
Uric acid, mg/dL	4.4 ± 1.0	2.40–8.50
Microalbuminuria, *μ*g/min	2.7 ± 0.7	0.5–89.8
Cortisol, *μ*g/day	41.0 ± 1.7	6.0–105.0

Values are means ± SD. Hb A1c, hemoglobin A(1c); TC, total cholesterol; HDL-C, high-density lipoprotein cholesterol; LDL-C, low-density lipoprotein cholesterol; Apo A1, apolipoprotein A1; Apo B, apolipoprotein B100; ALT, alanine aminotransferase; AST, aspartate aminotransferase; hs-CRP, high-sensitive C-reactive protein; IL-6, interleukin-6; TNF-*α*, tumor necrosis factor-alpha; PAI-1, plasminogen activator inhibitor-1.

**Table 3:** Independent contributions (standardized beta coefficients) of anthropometric and body composition variables to liver-to-spleen ratio, adjusted for age and BMI.

	Liver-to-spleen ratio	Percentage of variance explained** (%)
WC, cm	−0.229	7.7^#^
HC, cm	0.125	7.0^#^
WHR	−0.145	7.9^#^
SD, cm	−0.383*	10.1^#^
TFM, kg	−0.221	7.4^#^
TBFM, kg	−0.111	6.5^#^
TBLM, kg	−0.077	6.7^#^

All variables were entered in the regression models as continuous variables. Age did not present any independent association with the anthropometric studied variables.

** Variance explained by age, BMI, and the studied variable.

^#^ Independent associations of BMI*, P *< 0.01.

*P < 0.05.

^†^ *P* < 0.01.

^‡^ *P* < 0.001.

**Table 4:** Independent contributions (standardized beta coefficients) of abdominal adipose tissue depots and thigh body composition compartments to liver-to-spleen ratio, adjusted for age and BMI.

	Liver-to-spleen ratio	Percentage of variance explained** (%)
TAAT, cm^2^	−0.234	8.4
Ab SAT, cm^2^	−0.136	6.8
Superficial, cm^2^	0.014	6.3
Deep, cm^2^	−0.133	7.3
VAT, cm^2^	−0.241*	9.2
Mid-thigh AT, cm^2^	0.148	7.1
Mid-thigh SAT, cm^2^	0.129	7.1
Mid-thigh SFAT, cm^2^	0.295^†^	12.5
Muscle, cm^2^	0.005	6.2
HDM, cm^2^	−0.036	6.4
LDM, cm^2^	0.145	7.3

All variables were entered in the regression models as continuous variables. While BMI revealed independent associations with all body composition variables (*P *< 0.01), age did not present any independent relation with the studied variables.

** Variance explained by age, BMI, and the studied variable.

*P < 0.05.

^†^ *P* < 0.01.

**Table 5:** Independent contributions (standardized beta coefficients) of metabolic syndrome components to liver-to-spleen ratio, adjusted for age and BMI.

	Liver-to-spleen ratio	Percentage of variance explained** (%)
Glucose homeostasis		
Fasting insulin, *μ*IU/mL	−0.218*	10.1
Fasting glycemia, mg/dL	−0.069	6.6
Hb A1c, %	0.001	6.2
Lipid profile		
TC, mg/dL	−0.067	6.5
HDL-C, mg/dL	0.107	7.2
LDL-C, mg/dL	−0.021	6.2
TC/HDL-C ratio	−0.284^‡^	13.1
LDL-C/HDL-C ratio	−0.181*	9.0
Apo A1/Apo B100 ratio	0.067	6.5
Triglycerides, mg/dL	−0.257^†^	11.9
Blood pressure		
Systolic, mmHg	0.047	6.6
Diastolic, mmHg	0.177*	9.4
Liver enzymes		
ALT, IU/L	−0.437^‡^	24.8
AST, IU/L	−0.346^‡^	17.8
Inflammation		
hs-CRP, mg/dL	−0.006	3.6
Cytokines		
IL-6, pg/mL	−0.054	6.1
TNF-*α*, pg/mL	0.013	6.1
Hypercoagulation		
PAI-1, ng/mL	−0.208*	9.7
Fibrinogen, mg/dL	−0.011	6.3
Adipokines		
Leptin, ng/mL	−0.085	6.8
Adiponectin, ng/mL	0.041	6.4
Urine		
Uric acid, mg/dL	−0.178*	8.9
Microalbuminuria, *μ*g/min	0.071	6.7
Cortisol, *μ*g/day	−0.096	7.1

All variables were entered in the regression models as continuous variables. While age did not present any independent relation to the studied variables, BMI revealed an independent association with all metabolic syndrome features (*P* < 0.01).

** Variance explained by age, BMI and the studied variable.

*P < 0.05.

^†^ *P* < 0.01.

^‡^ *P* < 0.001.
